# Diversity and Community Structure of Bacteria in High-Altitude Proglacial Lakes in Southern Qinghai-Xizang Plateau

**DOI:** 10.3390/microorganisms14071398

**Published:** 2026-06-24

**Authors:** Yanyan Zheng, Dorji Phurbu

**Affiliations:** Key Laboratory of Plateau Fungi, Institute of Plateau Biology of Xizang Autonomous Region, Lhasa 850000, China

**Keywords:** 16S *rRNA*, physicochemical factors, beta diversity, proglacial environment, Tibetan Plateau

## Abstract

The proglacial lakes of the Qinghai-Xizang Plateau serve as natural laboratories for studying microbial adaptation to extreme environments. However, research on the composition and functional characteristics of microorganisms in these settings remains limited. In this study, three typical high-altitude proglacial lakes in southern Xizang (Qudengnima proglacial lake, Gangbugou proglacial lake, and Qiangyong proglacial lake) were selected as research subjects. Bacterial community structure, diversity in the water and sediment of these lakes were analyzed using 16S *rRNA* sequencing. The results showed that Pseudomonadota, Actinomycetota, and Bacteroidota were highly abundant across all samples. The relative abundances of Cyanobacteriota and Acidobacteriota, however, exhibited distinct habitat preferences: Cyanobacteriota was enriched in the water, whereas Acidobacteriota was predominantly found in sediment. Alpha diversity analysis showed that both species diversity and richness in Qiangyong proglacial lake were significantly higher than those in the other proglacial lakes, and within the same lake, both diversity and richness in sediment were higher than in the water. Beta diversity analysis indicated that the bacterial community structures in sediment were similar across different proglacial lakes, whereas those in water varied considerably among the lakes. LEfSe analysis identified 94 biomarkers that exhibited significant differences among the different proglacial lake environments at an LDA score threshold of 4. Redundancy analysis revealed that pH, total phosphorus, and ammonium nitrogen were the physicochemical factors significantly influencing the bacterial community structure in the water, while total carbon was the key driver for the community in sediments. This study preliminarily characterized the bacterial community structure and diversity in high-altitude proglacial lakes on the southern Qinghai-Xizang Plateau, which lays a theoretical foundation for exploiting microbial resources and understanding their ecological functions in such extreme environments.

## 1. Introduction

Glaciers are distinct ecosystems formed through dynamic processes such as long-term freeze–thaw cycles, accumulation, and compaction of snow, comprising three interrelated microenvironments: snow, ice, and meltwater. These extreme conditions, characterized by persistent low temperatures, nutrient scarcity, and high-intensity radiation, give rise to a unique proglacial ecosystem [1]. A study of 38 glaciers on the Qinghai-Xizang Plateau indicates that the multiyear average air temperature ranges from −13.4 to 2.9 °C and the multiyear average precipitation ranges from 76.9 to 927.8 mm. The DOC concentrations in ice core samples ranged from 0.005 to 5.05 mg L^−1^ with an average value of 0.54 ± 0.38 mg L^−1^, the TN concentrations ranged from 0.001 to 1.15 mg L^−1^ with an average value of 0.24 ± 0.16 mg L^−1^. The DOC concentrations ranged from 0.08 to 9.0 mg L^−1^ (0.89 ± 1.05 mg L^−1^), from 0.12 to 11.65 mg L^−1^ (1.19 ± 1.78 mg L^−1^), and from 0.05 to 16.15 mg L^−1^ (0.72 ± 1.71 mg L^−1^) in surface ice, surface snow, and snow pits, respectively. The TN concentration ranged from 0.01 to 1.88 mg L^−1^ (0.19 ± 0.22 mg L^−1^), from 0.07 to 3.06 mg L^−1^ (0.34 ± 0.35 mg L^−1^), and from 0.02 to 0.84 mg L^−1^ (0.15 ± 0.15 mg L^−1^) in surface ice, surface snow, and snow pits, respectively [2]. Furthermore, regarding solar radiation, there is considerable spatial heterogeneity across the Qinghai-Xizang Plateau. Overall, the annual mean shortwave radiation over Qinghai-Xizang Plateau glacierized regions is estimated to be 200–300 W m^−2^, exhibiting a distinct seasonal pattern with lower values during the cold season and higher values during the warm season [3]. The low temperature, nutrient scarcity, high-UV input constitutes a critical selective pressure for microbial communities in these extreme habitats.

Proglacial ecosystems harbor a rich microbial diversity [4]. For instance, in the Kuoqionggangri Glacier system on the Qinghai-Xizang Plateau, the proglacial ecosystem shows a Shannon diversity index exceeding 4, which is significantly higher than the corresponding values for supraglacial and subglacial habitats [5]. Across seven glacial lakes on the Qinghai-Xizang Plateau, Shannon diversity index of bacteria ranged from 3.67–6.00 in the water and from 6.88–9.06 in the sediment [6]. On the proglacial lakes of the LXZ Glacier, Shannon diversity index of bacteria exceeds 6, indicating a relatively rich diversity [7]. High-throughput sequencing analysis of proglacial soils has revealed that these environments harbor diverse microbial communities, including bacteria, fungi, archaea, and microfauna. The bacterial community comprises multiple phyla such as Proteobacteria, Acidobacteria, Bacteroidetes, Actinobacteria, Firmicutes, and Cyanobacteria [4]. As extremophiles, these microorganisms play a pivotal role in sustaining the ecological functions of the cryosphere—for instance, by engaging in processes such as carbon [8] and nitrogen cycling [9,10]. Moreover, certain microbial taxa or community-level traits can serve as indicators of climate change [11]. In the Geladaindong ice core on the Qinghai-Xizang Plateau, bacterial abundance varies in response to climatic conditions; correlation analyses show that it correlates positively with both δ^18^O (a temperature/precipitation-source proxy) and Ca^2+^ (a dust/mineral-aerosol proxy), i.e., higher temperatures and more frequent dust deposition both elevate bacterial loading in the ice [12]. By contrast, in the Malan ice core—a 102-m-long, centennial–multi-centennial record—microbial concentration is inversely related to temperature over longer timescales and, to some extent, positively correlated with mineral concentrations; in other words, higher microbial loads mark colder periods with stronger dust-transport regimes, whereas lower loads correspond to warmer periods with weaker/less-frequent dust events [13]. Complementarily, across 22 proglacial lakes on the Qinghai–Xizang Plateau, the mean annual temperature emerges as the primary driver of bacterioplankton biodiversity—increasing richness but decreasing β-diversity—with cold-preferred taxa declining as temperature rises [14]. Climate warming leads to glacier melting, with meltwater contributing to the formation of proglacial lakes [15]. This also results in the degradation of the glacial microorganisms’ habitat, causing them to be continuously released from the glacier and transported into proglacial lakes via meltwater [5,16,17,18]. As lakes directly or indirectly influenced by glaciers, proglacial lakes are more sensitive responders to climate change compared to other lake types and serve as ideal recorders of climatic variations. Their microbial communities can preserve a record of historical climate change processes. Therefore, investigating the microbial community structure in proglacial lakes is crucial for exploiting proglacial microbial resources, assessing the health of proglacial ecosystems, and understanding climate change feedbacks. As the “Asian Water Tower”, the Qinghai-Xizang Plateau hosts an abundance of mid- and low-latitude glaciers. Since the Last Deglaciation, glaciers in this region have been persistently retreating, giving rise to a series of proglacial lakes that preserve a continuous record of glacier–climate interactions [19,20]. And the microbial communities within them document historical climate change and ecosystem succession, thereby serving as ideal indicators for studying global environmental change [21]. Currently, research on glaciers and proglacial lakes in the Qinghai-Xizang Plateau has primarily focused on geology, remote sensing, and meteorology [22,23,24]. Studies on microorganisms in this region are predominantly found in environmental matrices such as proglacial ice cores and soils [25,26], whereas relatively few studies have been reported on microorganisms in proglacial lakes formed by proglacial meltwater [6]. Previous bacteriological studies in high-altitude lakes on the Qinghai–Xizang Plateau have provided valuable insights into bacterial community patterns and their environmental drivers. Xing et al. investigated the bacterioplankton composition in several high-altitude lakes in the eastern plateau and found remarkably low taxon richness, with community variation primarily explained by salinity [27]. In a study focused on a single proglacial lake, Gu et al. [28] conducted a five-month-long characteristic analysis of the bacterial community in the water body of the Strongy Ice-covered Lake. The results indicated that the dynamic of bacterial community in the proglacial stream and lake water is influenced by environmental factors, and the community composition assembly of the Qiangyong glacial stream and lake could be dynamic and primarily governed by deterministic processes. Complementing these water-focused works, Xiong et al. examined bacterial communities in sediments across multiple alkaline lakes on the plateau and demonstrated that geographic distance and pH jointly governed bacteriacommunity turnover, highlighting the importance of both dispersal limitation and local environmental filtering [29]. Together, these studies underscore that bacterial communities in Qinghai-Xizang Plateau lakes are sensitive to a combination of environmental and spatial factors. This study selected three high-altitude proglacial lakes in Xizang as the study sites: Qudengnima proglacial lake, Gangbugou proglacial lake, and Qiangyong proglacial lake. These areas are less affected by human activities, which facilitates the investigation of microbial community structures in proglacial lake environments under near-natural conditions. Regarding the microbial communities in Qiangyong proglacial lake, only one study has been reported in the literature [28], while no reports are currently available on the microbial communities in Qudengnima proglacial lake and Gangbugou proglacial lake. Consequently, significant knowledge gaps remain in our understanding of microbial life in these proglacial extreme environments. First, baseline data on microbial diversity and community composition are entirely lacking for most proglacial lakes. Second, it is unknown whether the limited patterns observed in a single proglacial lake are representative of the wider region. Third, the lack of comparative data between water and sediment habitats within these proglacial lakes hinders our understanding of habitat-driven community differentiation in extreme proglacial environments. To fill these gaps, we address the following research questions: (1) What are the similarities and differences in microbial community structure and diversity among the three proglacial lakes? (2) Which environmental variables best explain the observed variation in community composition? (3) How do microbial community compositions differ between the water and sediment habitats within these proglacial lakes in the southern Qinghai-Xizang Plateau, and what factors drive these differences?

Compared with previous work, which has been restricted to a single proglacial lake and/or a single habitat type, the novelty of this study lies in the simultaneous comparison of bacterial communities across multiple proglacial lakes and between water and sediment habitats in the southern Qinghai–Xizang Plateau. This design allows us to disentangle lake-specific versus habitat-specific effects on community structure, and to identify key environmental drivers at a regional scale—insights that cannot be obtained from single-lake studies. In this study, 16S *rRNA* gene amplicon sequencing was employed to analyze the bacterial community structures in the water and sediments of the aforementioned three proglacial lakes. The aims were to compare the differences in bacterial taxonomic composition among different lakes and environmental media, and to identify the key physicochemical parameters influencing the community structures. This work is intended to lay a foundation for microbial research in the glacial ecosystems of the Qinghai-Xizang Plateau, and to provide a scientific basis for the subsequent development and utilization of glacial microbial resources as well as for investigating the response of glacial microorganisms to environmental change.

## 2. Materials and Methods

### 2.1. Study Area and Sampling

The Qudengnima glacier is located in Gamba County, Shigatse, Xizang Autonomous Region, and serves as the water source for the “Xizang Holy Water”. The Qudengnima proglacial lake (approximate coordinate was 28°1′ N, 88°15′ E) at the glacier terminus has an altitude of approximately 5200 m. The Gangbugou glacier is situated in Nagarzê County, Shannan, Xizang Autonomous Region. Its meltwater forms the Gangbugou proglacial lake (approximate coordinate was 28°56′ N, 90°12′ E), with a lake surface elevation of approximately 4900 m. The Qiangyong glacier is located in Nagarzê County, Shannan, Xizang Autonomous Region. Its meltwater feeds the Qiangyong proglacial lake (approximate coordinate was 28°52′ N, 90°14′ E), which is situated on the northern slope of the glacier at an elevation of approximately 4870 m and lies within 1 km of the glacier’s terminal snout [22]. In July 2024, three sampling sites were established at Qudengnima proglacial lake, Gangbugou proglacial lake, and Qiangyong proglacial lake, respectively, based on the distribution of water bodies. Water and sediment samples were collected simultaneously at each site, resulting in a total of nine sampling locations (the sampling site locations for each proglacial lake are shown in Table 1). Each sample was mixed from three replicates collected at the same site. Water samples were collected using a sterile water sampler (Youlisheng, Shenzhen, China), with three biological replicates per sample. Each sample was divided into two portions. One portion was placed in a sterile plastic bucket and was used for vacuum filtration with mixed cellulose ester membranes (0.22 μm pore size, 47 mm diameter; Merck Millipore, Darmstadt, Germany; Made in Ireland). Water samples were immediately placed in a portable refrigerator (Dometic, Solna, Sweden) at 4 °C and transported to the hotel for filtration. A volume of 2000 mL of water was filtered per membrane. The filtered membranes were placed into 2 mL sterile centrifuge tubes (NEST, Wuxi, China) and stored in a portable refrigerator freezer at −20 °C. The other portion was stored at 4 °C for subsequent determination of physicochemical factors. Sediment samples were collected using a grab sampler (Jienuobang, Nanjing, China) and collected from a layer approximately 15 cm deep. Three biological replicates were taken for each sample. Approximately 150 g of sediment was collected for each sample and divided into two portions: one was placed into a sterile centrifuge tube (NEST, Wuxi, China) and stored in a portable refrigerator freezer at −20 °C, while the other was air dried for subsequent determination of physicochemical factors. At the end of the sampling, all samples were transported back to the laboratory. The samples required for high-throughput sequencing are stored at −80 °C until DNA extraction is carried out, while the samples for measuring physicochemical factors are immediately tested.

Water samples from Qudengnima proglacial lake were designated QDNM-W1, QDNM-W2, and QDNM-W3, while sediment samples were designated QDNM-S1, QDNM-S2, and QDNM-S3. Samples from Gangbugou proglacial lake included water samples GBG-W1, GBG-W2, and GBG-W3, along with sediment samples GBG-S1, GBG-S2, and GBG-S3. For Qiangyong proglacial lake, the water samples were designated QY-W1, QY-W2, and QY-W3, and the sediment samples were designated QY-S1, QY-S2, and QY-S3.

### 2.2. Determination of Physicochemical Factors

The physicochemical factors of water samples were measured as follows: pH, electrical conductivity (EC), dissolved oxygen (DO), oxidation–reduction potential (ORP), and water temperature (T) were measured in situ using a Hach portable water quality meter (Hach, Loveland, CO, USA). Organic carbon (OC) was determined using a TOC analyzer (Shimadzu TOC-VCPH; Shimadzu, Kyoto, Japan) with 0.45 μm membrane filtration. Total nitrogen (TN) and total phosphorus (TP) were digested with potassium persulfate (GHTECH, Shantou, China) and quantified by ultraviolet spectrophotometry (Shimadzu UV-2450; Shimadzu, Kyoto, Japan). Ammonium nitrogen (NH_4_^+^-N), nitrate nitrogen (NO_3_^−^-N), and nitrite nitrogen (NO_2_^−^-N) were determined using a continuous flow analyzer (SEAL-AA3 Auto Analyzer; SEAL Analytical, Norderstedt, Germany).

The physicochemical factors of sediment samples were determined as follows: total nitrogen (TN) and total carbon (TC) were determined by elemental analysis using a FlashSmart elemental analyzer (Thermo Fisher Scientific, Waltham, MA, USA). Total phosphorus (TP) was quantified using a Shimadzu UV-1900i spectrophotometer (Shimadzu, Kyoto, Japan) via the molybdenum-antimony ascorbic acid reduction method following NaOH (GHTECH, Shantou, China) fusion pretreatment. Ammonium nitrogen (NH_4_^+^-N) and nitrate nitrogen (NO_3_^−^-N) were extracted with potassium chloride (Sinopharm, Shanghai, China) and determined using a continuous flow analyzer. The pH was measured potentiometrically using a Sartorius PB-10 pH meter (Sartorius, Göttingen, Germany). Organic carbon (OC) was determined by the potassium dichromate (Xilong, Shantou, China)–concentrated sulfuric acid (Kelong, Chengdu, China) high-temperature digestion method using a Brand Titrette titrator (Brand, Wertheim, Germany). Total sulfur (TS) was determined by the barium sulfate (Kelong, Chengdu, China) turbidimetric method using a Shimadzu UV-2450 spectrophotometer. Total dissolved solids (TDS) was determined by the oven-drying gravimetric method (oven: Fuma, Shanghai, China). Electrical conductivity (EC) was measured using a DDSJ-308F conductivity meter (Leici, Shanghai, China) after shaking and centrifugation. Moisture content (MC) was determined by the oven drying method.

### 2.3. DNA Extraction, PCR Amplification, and Illumina Sequencing

The genomic DNA of microorganisms on filter membranes was extracted using the QIAamp^®^ DNA Micro Kit (QIAGEN, Hilden, Germany), while that in the sediment samples was extracted using the DNeasy^®^ PowerSoil^®^ Pro Kit (QIAGEN, Hilden, Germany). The concentration and purity of the DNA were determined using a NanoDrop 2000 spectrophotometer (Thermo Fisher Scientific, Waltham, MA, USA), and its integrity was assessed by 1% agarose gel electrophoresis (Thermo Fisher Scientific, Waltham, MA, USA). We amplified the V3–V4 regions of the 16S *rDNA* gene from bacteria using barcoded primers 338F (5′-ACTCCTACGGGAGGCAGCAG-3′) and 806R (5′-GGACTACHVGGGTWTCTAAT-3′). PCR amplification was performed in a 20 μL reaction mixture including 1 μL of DNA template, 10 μL of 2 × Pro Taq Master Mix (AGbio, Changsha, China), 0.8 μL each of the forward and reverse primers (5 μM; Sangon, Shanghai, China), and ddH_2_O added to a final volume of 20 μL. The amplification program in the ABI GeneAmp^®^ 9700 thermal cycler (Thermo Fisher Scientific, Waltham, MA, USA) was performed as follows: initial denaturation at 95 °C for 3 min, followed by 27 cycles of denaturation at 95 °C for 30 s, annealing at 55 °C for 30 s, extension at 72 °C for 45 s, and a final extension at 72 °C for 10 min. The PCR product was verified on a 2% agarose gel (Thermo Fisher Scientific, Waltham, MA, USA), the target band was excised, and DNA was purified using the AxyPrep DNA Gel Extraction Kit (Axygen Biosciences, Union City, CA, USA). Amplicon concentration was determined fluorometrically using the QuantiFluor™-ST/Quantus Fluorometer (Promega, Madison, WI, USA). Sequencing libraries were constructed using the NEXTFLEX^®^ Rapid DNA-Seq Kit (Bioo Scientific, Austin, TX, USA). Purified amplicons were pooled in equimolar amounts and paired-end sequenced on an Illumina Nextseq2000 platform (Illumina, San Diego, CA, USA) according to the standard protocols by Majorbio Bio-Pharm Technology Co., Ltd. (Shanghai, China), generating 2 × 300 bp paired-end reads (PE300). The paired-end reads obtained from sequencing were assembled using FLASH v1.2.11 [30] based on overlapping regions. Quality control and filtering of the sequences were performed using Fastp v0.19.6 [31]. Operational taxonomic units (OTUs) were clustered using Uparse v11.0.667 [32,33] with a 97% sequence similarity threshold, and taxonomic annotation of the OTUs was conducted based on the Silva database (v138.2) [34].

### 2.4. Statistical Analyses

The Shapiro–Wilk test was performed to assess data normality. One-way analysis of variance (ANOVA) and Duncan’s multiple range test were performed using SPSS v27.0 (IBM Corp., Armonk, NY, USA) [35], and data visualization was conducted with Origin 2021 (OriginLab Corporation, Northampton, MA, USA) [36]. Community composition bar plots and Venn diagrams were generated in R v4.3.2 [37]. Alpha diversity analysis was performed using Mothur v1.48.5 [38], while beta diversity distances were calculated with Qiime v1.91 [39] (https://doi.org/10.5281/zenodo.18036) and further analyzed/plotted using the vegan package (v2.6.10) in R v4.3.2. Differences in bacterial α-diversity indices between sediment and water habitats were tested using independent-samples *t*-test. Linear discriminant analysis (LDA) was performed with LEfSe tool (http://galaxy.biobakery.org/, accessed on 5 March 2026) [40] to identify differentially abundant taxa among groups. Redundancy analysis (RDA) and corresponding plotting were performed with the vegan package (v2.6.10) in R v4.3.2 [41].

## 3. Results

### 3.1. Physicochemical Properties of Samples

**Figure 1:**
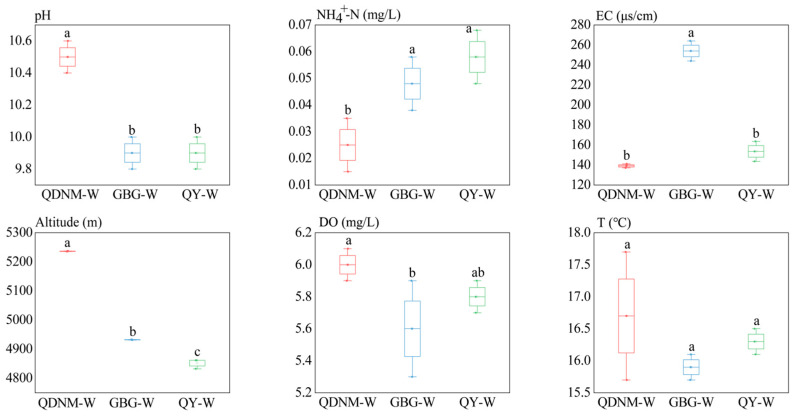
Physicochemical properties of water in different proglacial lakes. Note: The samples marked with different lowercase letters showed significant differences (*p* < 0.05), and the least significant difference (LSD) test was used. Abbreviations: NH_4_^+^-N, ammonium nitrogen; EC, Electrical Conductivity; DO, Dissolved Oxygen; T, Temperature; QDNM-W, water samples from Qudengnima proglacial lake; GBG-W, water samples from Gangbugou proglacial lake; QY-W, water samples from Qiangyong proglacial lake.

Differences in the physicochemical properties of water and sediment samples from the three proglacial lakes are shown in Figure 1 and Figure S1. Among the 12 physicochemical parameters of water samples, with the exception of TN and T, all other parameters exhibited significant differences among the different proglacial lakes (*p* < 0.05). Among the three proglacial lakes, Qudengnima exhibited the highest values for pH, altitude, DO, NO_2_^−^-N, and OC. The highest conductivity was recorded in Gangbugou, while the highest levels of NH_4_^+^-N, NO_3_^−^-N, ORP, and TP were recorded in Qiangyong. Differences in the physicochemical properties of water and sediment samples from the three proglacial lakes are shown in Figure S2. TN, TP, pH, OC, TDS, MC, and altitude exhibited significant differences across the different proglacial lakes (*p* < 0.05). The concentrations of TN, TP, OC, and MC ranged from 0.15 to 0.34 g/kg, 0.37 to 0.86 g/kg, 2.84 to 12.13 g/kg, 10.59% to 19.51%, respectively, with the highest values all observed in Qiangyong proglacial lake. The pH ranged from 8.61 to 9.04, indicating an alkaline condition, and the highest value was recorded in Qudengnima proglacial lake. The TDS concentration ranged from 0.05% to 0.50%, and was significantly higher in Qudengnima proglacial lake than in Gangbugou proglacial lake and Qiangyong proglacial lake.

### 3.2. Bacterial Diversity

#### 3.2.1. Analysis of OTU

**Figure 2:**
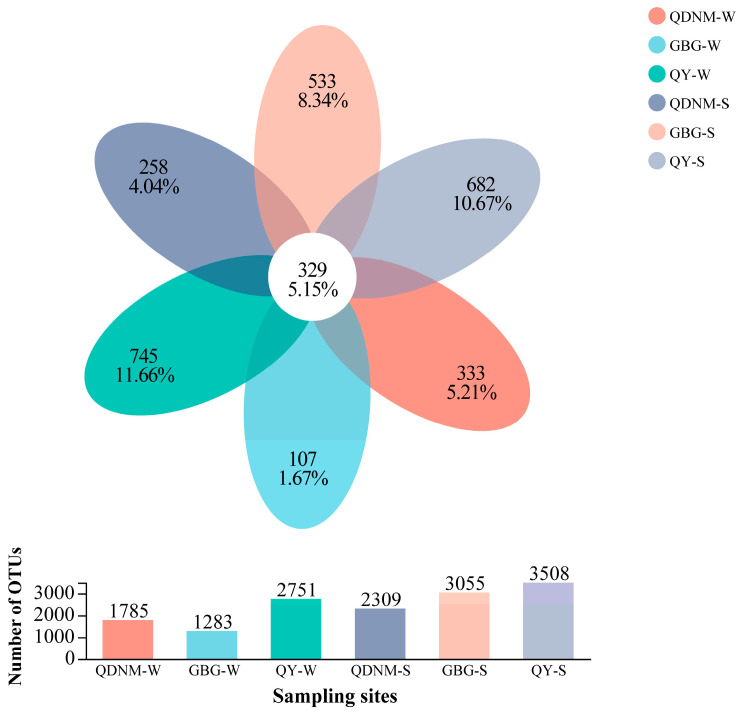
Venn diagram of water and sediment at the OTU level in different proglacial lakes. Note: In the petal diagram, the number in the central overlapping area indicates the number of core OTUs shared by all groups, the numbers on the petals represent the number of unique OTUs in each group, the percentages below the numbers indicate the proportion of unique OTUs in that group to the total number of OTUs. In the bar chart, the numbers represent the total number of observed OTUs for each sample. Abbreviations: OTU, Operational Taxonomic Unit; QDNM-W, water samples from Qudengnima proglacial lake; GBG-W, water samples from Gangbugou proglacial lake; QY-W, water samples from Qiangyong proglacial lake; QDNM-S, sediment samples from Qudengnima proglacial lake; GBG-S, sediment samples from Gangbugou proglacial lake; QY-S, sediment samples from Qiangyong proglacial lake.

A total of 14,691 OTUs were obtained, with 4094, 4338, and 6259 derived from the Qudengnima proglacial lake, Gangbugou proglacial lake, and Qiangyong proglacial lake, respectively. The Venn diagram based on OTU classification (Figure 2) showed that 329 OTUs (5.15% of the total) were shared among the water and sediment samples from the three proglacial lakes. The numbers of unique OTUs in the water samples from Qudengnima proglacial lake, Gangbugou proglacial lake, and Qiangyong proglacial lake were 333 (5.21%), 107 (1.67%), and 745 (11.66%), respectively, while those in the sediment samples were 258 (4.04%), 533 (8.34%), and 682 (10.67%), respectively. Qiangyong proglacial lake exhibited the highest number of unique OTUs, suggesting that it may harbor a more distinct habitat compared to the other two proglacial lakes.

#### 3.2.2. Alpha Diversity Analysis

The Coverage index was used to evaluate the representativeness of the sequencing results for the microbial diversity within the samples. The coverage of all sample libraries in this study exceeded 98.4%, indicating that the sequencing depth was sufficient to capture the majority of bacterial communities present and that the results reliably reflect the actual composition of the bacterial assemblages.

**Figure 3:**
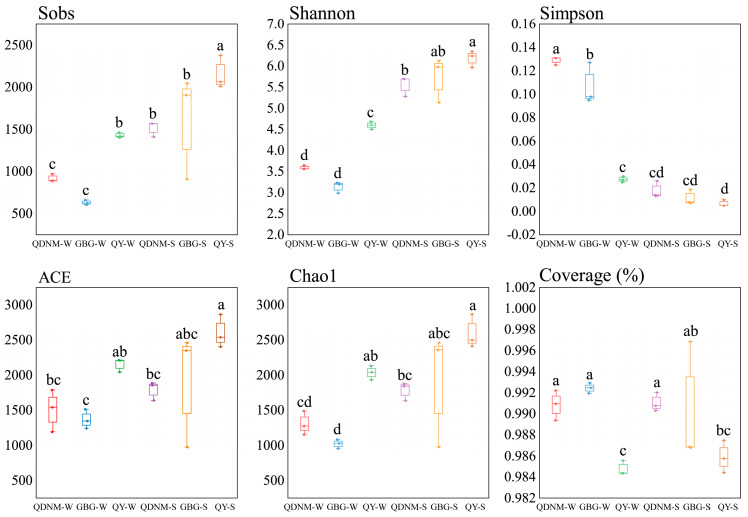
Diversity and richness of bacterial community in different proglacial lakes. Note: The samples marked with different lowercase letters showed significant differences (*p* < 0.05), and the least significant difference (LSD) test was used. Abbreviations: Sobs, Observed Species; Shannon, Shannon–Wiener diversity index; Simpson, Simpson’s diversity index; ACE, Abundance-based Coverage Estimator; Chao 1, Chao 1 Richness Estimator; Coverage, Good’s Coverage; QDNM-W, water samples from Qudengnima proglacial lake; GBG-W, water samples from Gangbugou proglacial lake; QY-W, water samples from Qiangyong proglacial lake; QDNM-S, sediment samples from Qudengnima proglacial lake; GBG-S, sediment samples from Gangbugou proglacial lake; QY-S, sediment samples from Qiangyong proglacial lake.

For alpha diversity analysis, commonly used diversity indices were selected, including the Chao1 index, ACE index, Shannon index, and Simpson index. Species richness was assessed using the first two indices, while species diversity was characterized by the latter two. The Shannon index is positively correlated with species diversity, whereas the Simpson index shows a negative correlation. Based on OTU sequences, the alpha diversity indices of water and sediment from different sampling sites were calculated (Figure 3). The Shannon index ranged from 2.99 to 6.35, with the sediment sample from Qiangyong proglacial lake showing the highest value. The Simpson index ranged from 0.005 to 0.131. The Chao1 and ACE indices ranged from 957.59 to 2871.17 and from 976.66 to 2866.42, respectively, both peaking in the sediment sample from Qiangyong proglacial lake. The observed species (Sobs) index ranged from 606 to 2382, and the Coverage ranged from 98.4% to 99.7%. Overall, both species diversity (*p* < 0.001) and species richness (*p* < 0.05) were higher in the sediments than in the water across the three proglacial lakes. In terms of water samples, Qiangyong proglacial lake exhibited significantly higher species diversity and richness compared to Qudengnima proglacial lake and Gangbugou proglacial lake. Similarly, among the sediment samples, Qiangyong proglacial lake showed the highest values for both indices.

#### 3.2.3. Beta Diversity Analysis

**Figure 4:**
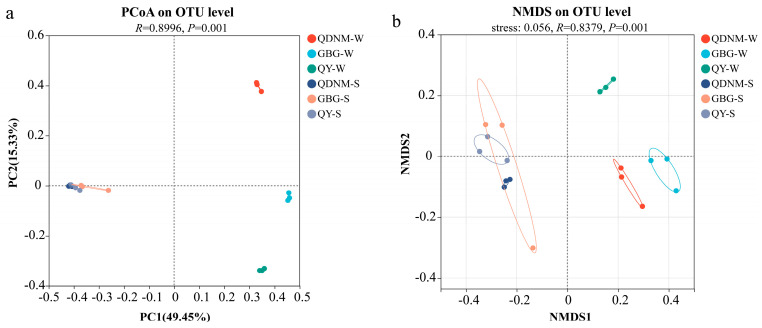
The principal coordinate analysis (**a**) and non-metric multidimensional scaling analysis (**b**) of bacterial communities in different proglacial lakes. Abbreviations: PCoA, Principal Coordinate Analysis; OTU, Operational Taxonomic Unit; NMDS, Non-metric Multidimensional Scaling; PC1, Principal Coordinate 1; PC2, Principal Coordinate 2; NMDS1, Non-metric Multidimensional Scaling Axis 1; NMDS2, Non-metric Multidimensional Scaling Axis 2; *R*, R-value; *P*, *p*-value; stress, stress value; QDNM-W, water samples from Qudengnima proglacial lake; GBG-W, water samples from Gangbugou proglacial lake; QY-W, water samples from Qiangyong proglacial lake; QDNM-S, sediment samples from Qudengnima proglacial lake; GBG-S, sediment samples from Gangbugou proglacial lake; QY-S, sediment samples from Qiangyong proglacial lake.

Principal coordinate analysis (PCoA) was performed based on the Bray–Curtis distance algorithm. Figure 4a shows that PC1 (49.45%) and PC2 (15.33%) together explained 64.78% of the microbial community variation (ANOSIM: *R* = 0.900, *p* = 0.001). Non-metric multidimensional scaling (NMDS) was conducted based on the Unweighted UniFrac distance algorithm, with a stress value of 0.056 (<0.1), indicating a good ordination. Thus, the plot reliably represents the similarity of community structures among samples, and statistical analysis revealed significant differences between samples (ANOSIM: *R* = 0.838, *p* = 0.001). The PCoA (Figure 4a) and NMDS (Figure 4b) yielded consistent results: bacterial communities in the water and sediment samples of the three proglacial lakes clustered separately, indicating that the bacterial community composition differed among the six types of samples. The sediment samples from the three proglacial lakes clustered closely in the ordination plot, indicating relatively similar bacterial community compositions with minor differences. In contrast, the water samples were more dispersed, suggesting greater distinctions in bacterial species composition among the three proglacial lakes.

**Figure 5:**
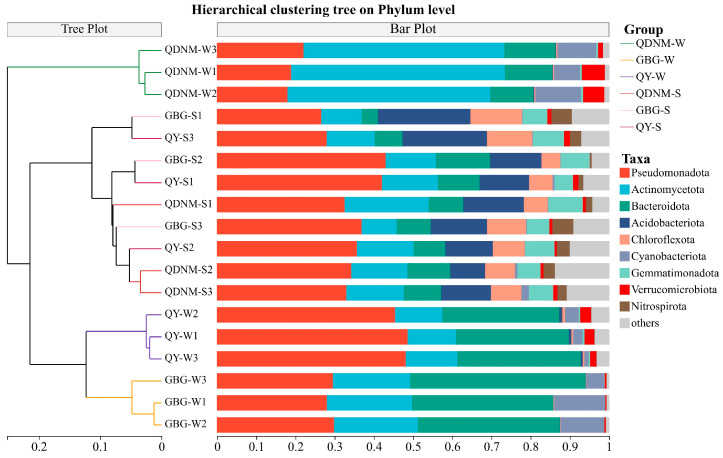
UPGMA hierarchical cluster analysis of bacterial communities in different proglacial lakes. The “others” category includes phyla with an average relative abundance below 1%. Abbreviations: QDNM-W, water samples from Qudengnima proglacial lake; GBG-W, water samples from Gangbugou proglacial lake; QY-W, water samples from Qiangyong proglacial lake; QDNM-S, sediment samples from Qudengnima proglacial lake; GBG-S, sediment samples from Gangbugou proglacial lake; QY-S, sediment samples from Qiangyong proglacial lake.

UPGMA hierarchical clustering analysis was performed using the Bray–Curtis distance algorithm at the phylum level (Figure 5). A dendrogram was constructed based on sample clustering and combined with stacked bar plots showing the complete phylum-level community composition. The results revealed distinct clustering patterns between water and sediment samples. Specifically, water samples from the three proglacial lakes (Qudengnima, Gangbugou, and Qiangyong) each formed separate, lake-specific clusters, indicating that the bacterial community composition in the water is unique to each lake. In contrast, sediment samples did not show clear lake-specific clustering; instead, they were more intermixed, suggesting that sedimentary bacterial communities are less differentiated among the three proglacial lakes. Overall, these results confirm habitat-dependent differences in bacterial community structure and reveal that water samples are more lake-specific than sediment samples in these proglacial lakes.

**Figure 6:**
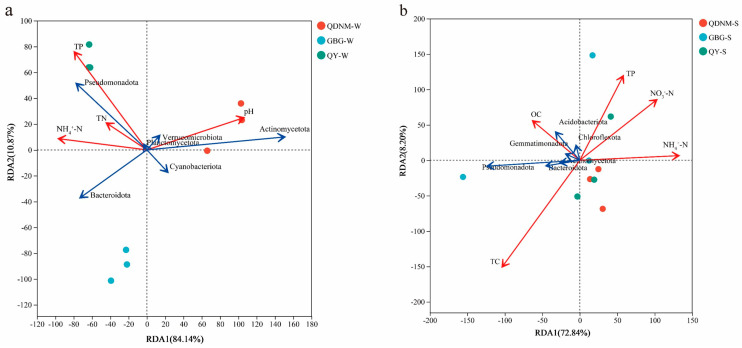
Redundant analysis (RDA) of bacterial dominance phylum and physicochemical parameters of water (**a**) and sediment (**b**) in different proglacial lakes. Abbreviations: RDA1, Redundancy Analysis axis 1; RDA2, Redundancy Analysis axis 2; TP, Total Phosphorus; TN, Total Nitrogen; TC, Total Carbon; NH_4_^+^-N, Ammonium Nitrogen; NO_3_^−^-N, Nitrate Nitrogen; OC, Organic Carbon; QDNM-W, water samples from Qudengnima proglacial lake; GBG-W, water samples from Gangbugou proglacial lake; QY-W, water samples from Qiangyong proglacial lake; QDNM-S, sediment samples from Qudengnima proglacial lake; GBG-S, sediment samples from Gangbugou proglacial lake; QY-S, sediment samples from Qiangyong proglacial lake.

To further identify the environmental drivers underlying these beta diversity patterns, we performed redundancy analysis (RDA). Physicochemical parameters with strong multicollinearity (VIF > 10) were excluded based on variance inflation factor (VIF) analysis, while those with low multicollinearity (VIF < 10) were retained for subsequent correlation analysis with the bacterial communities. For water samples, pH, TN, TP, and NH_4_^+^-N (with VIF values of 2.52, 1.64, 1.91, and 3.99, respectively) were retained. For sediment samples, TC, TP, NH_4_^+^-N, NO_3_^−^-N, and OC (with VIF values of 3.07, 4.51, 1.28, 4.24, and 4.88, respectively) were retained. Based on the detrended correspondence analysis (DCA) results, which showed axis lengths less than 3.0, redundancy analysis (RDA) was selected to examine the key physicochemical parameters influencing bacterial community variations in the three proglacial lakes. In the redundancy analysis (RDA) of water bacterial communities and physicochemical factors (Figure 6a), RDA1 and RDA2 explained 84.14% and 10.87% of the variation, respectively, cumulatively accounting for 95.01% of the total variance. In the redundancy analysis (RDA) of sediment bacterial communities and physicochemical factors (Figure 6b), RDA1 and RDA2 explained 72.84% and 8.20% of the variation, respectively, with a cumulative explanation of 81.04%. These results indicated that the analysis effectively captured the correlations between the physicochemical parameters and the bacterial communities. In the water, pH was positively correlated with Actinomycetota, Cyanobacteriota, Verrucomicrobiota, and Planctomycetota. Both TN and NH_4_^+^-N showed positive correlations with Bacteroidota and Pseudomonadota, while TP was positively correlated with Pseudomonadota. In the sediment, OC was positively correlated with dominant phyla such as Acidobacteriota, Gemmatimonadota, and Chloroflexota. NH_4_^+^-N showed negative correlations with Acidobacteriota and Gemmatimonadota, while both NO_3_^−^-N and TP were negatively correlated with Pseudomonadota, Bacteroidota, and Actinomycetota. The physicochemical parameters influencing bacterial communities in the water differed among the three proglacial lakes: pH was positively correlated with the bacterial community in Qudengnima proglacial lake, while TP showed a positive correlation with that in Qiangyong proglacial lake.

#### 3.2.4. LEfSe Analysis

**Figure 7:**
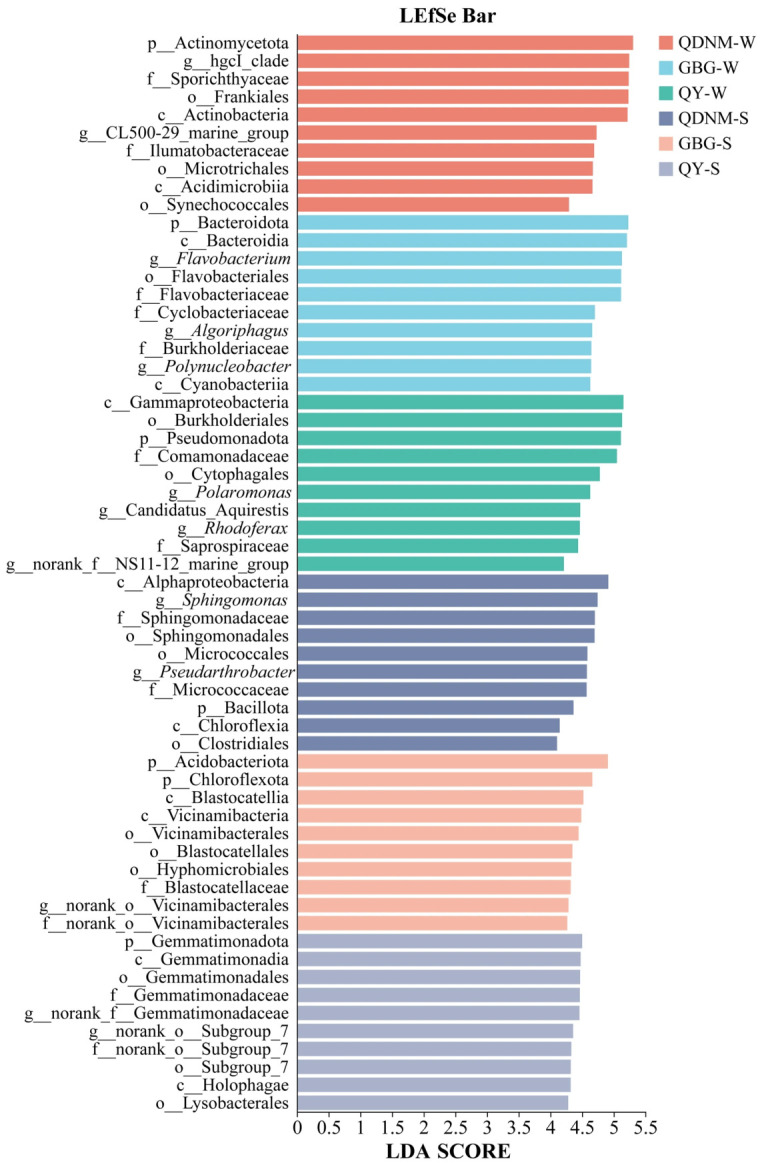
Bar chart of LDA value distribution of bacteria in different proglacial lakes (α = 0.05; LDA > 4). Abbreviations: LEfSe, Linear Discriminant Analysis Effect Size; LDA, Linear Discriminant Analysis; p, phylum; c, class; o, order; f, family; g, genus; QDNM-W, water samples from Qudengnima proglacial lake; GBG-W, water samples from Gangbugou proglacial lake; QY-W, water samples from Qiangyong proglacial lake; QDNM-S, sediment samples from Qudengnima proglacial lake; GBG-S, sediment samples from Gangbugou proglacial lake; QY-S, sediment samples from Qiangyong proglacial lake.

LEfSe (Linear Discriminant Analysis Effect Size) analysis was performed on the bacterial community composition in water and sediment samples from the three proglacial lakes to identify multilevel differences in taxa from phylum to genus (Figure 7 and Figure S3). At an LDA threshold of 3, a total of 323 biomarkers from the phylum to genus levels showed significant differences (*p* < 0.05). When the threshold was raised to 4, 94 biomarkers remained significantly different across the three proglacial lake (*p* < 0.05). Among the 94 significantly different biomarkers, there were 10 phyla, 15 classes, 19 orders, 25 families, and 25 genera. Actinomycetota and Bacillota were enriched in Qudengnima proglacial lake; Bacteroidota, Acidobacteriota, and Chloroflexota were enriched in Gangbugou proglacial lake; Pseudomonadota and Gemmatimonadota were enriched in Qiangyong proglacial lake. At the genus level, taxa such as hgcI_clade and *Sphingomonas* were more abundant in Qudengnima proglacial lake, whereas *Flavobacterium*, *Algoriphagus*, and *Polynucleobacter* were enriched in Gangbugou proglacial lake, and groups including *Polaromonas* and *Rhodoferax* were more prevalent in Qiangyong proglacial lake.

### 3.3. Bacterial Community Composition

**Figure 8:**
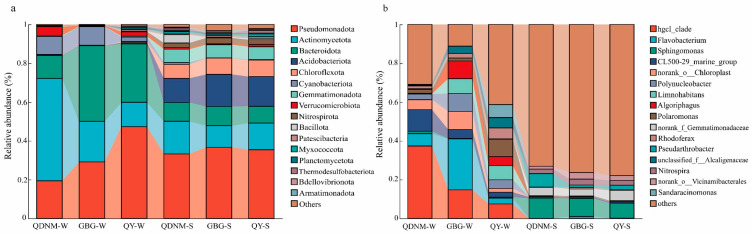
The composition of bacterial communities of water and sediment at the phylum level (**a**) and the genus level (**b**) in different proglacial lakes. Abbreviations: QDNM-W, water samples from Qudengnima proglacial lake; GBG-W, water samples from Gangbugou proglacial lake; QY-W, water samples from Qiangyong proglacial lake; QDNM-S, sediment samples from Qudengnima proglacial lake; GBG-S, sediment samples from Gangbugou proglacial lake; QY-S, sediment samples from Qiangyong proglacial lake.

The composition and abundance of bacterial communities at the phylum level in water and sediment samples from the three proglacial lakes are presented in Figure 8a. The results showed that the bacterial communities in the water of the three proglacial lakes were mainly composed of Pseudomonadota, Actinomycetota, Bacteroidota, Cyanobacteriota, and Verrucomicrobiota. The bacterial community structures at the phylum level were similar among the water samples from the different proglacial lakes, but the relative abundances of individual phyla varied considerably. Among them, Actinomycetota showed the highest relative abundance (52.74%) in the water of Qudengnima proglacial lake, Bacteroidota was most abundant (39.00%) in Gangbugou proglacial lake, and Pseudomonadota reached the highest relative abundance (47.35%) in Qiangyong proglacial lake. The bacterial community structures in sediments of the different proglacial lakes were similar, with relatively small differences in the relative abundances among the major phyla. The communities were mainly composed of Pseudomonadota, Actinomycetota, Bacteroidota, Acidobacteriota, Chloroflexota, Gemmatimonadota, and Nitrospirota. In the sediment samples of the three proglacial lakes, Pseudomonadota consistently exhibited the highest relative abundance (33.25–36.69%), with little variation among the lakes. This was followed by Actinomycetota and Acidobacteriota, with relative abundances of 16.83% and 12.43% in Qudengnima proglacial lake, 11.14% and 16.52% in Gangbugou proglacial lake, and 13.73% and 15.28% in Qiangyong proglacial lake, respectively. Compared to the water samples, the bacterial community composition in the sediment samples was more consistent, with less variation in the relative abundances of the dominant phyla. Moreover, a higher proportion of sequences were classified as “Others” in the sediment samples, indicating the presence of a greater number of low-abundance (rare) taxa.

The composition and abundance of bacterial communities at the genus level in water and sediment samples from the three proglacial lakes are presented in Figure 8b. At the genus level, hgcI_clade was the most relatively abundant taxon (37.36%) in the water of Qudengnima proglacial lake, while *Flavobacterium* (26.34%) and *Polaromonas* (9.00%) were most abundant in Gangbugou proglacial lake and Qiangyong proglacial lake, respectively. Moreover, the proportion of low-abundance species was highest in the water of Qiangyong proglacial lake compared to the other two lakes. Regarding the community composition in the sediments, *Sphingomonas* showed the highest relative abundance (7.80–10.38%) in three proglacial lakes. *Pseudarthrobacter* exhibited a relatively high abundance in Qudengnima proglacial lake, accounting for 6.91%, while the relative abundance of norank_f_Gemmatimonadaceae was similar across three lakes, ranging from 3.59% to 5.30%. The bacterial community composition and relative abundance exhibited greater variation in the water samples of the different proglacial lakes, whereas those in the sediment samples were relatively consistent. Moreover, the proportion of low-abundance taxa was substantially higher in the sediments than in the water.

## 4. Discussion

In this study, bacterial community structure and diversity in three proglacial lakes on the Qinghai-Xizang Plateau were investigated using 16S *rRNA* gene amplicon sequencing. Comparative analysis revealed that although these lakes were fed by high-altitude glaciers in southern Xizang and share certain consistent community features, certain differences in their bacterial compositions were also identified (Figure 8). As shown in Figure 8a, dominant bacterial phyla in the water of the three proglacial lakes included Pseudomonadota (syn. Proteobacteria), Actinomycetota, and Bacteroidota—a profile suggesting conserved community composition under the high-radiation, oligotrophic, and cold conditions of these glacial-fed proglacial lake habitats. Qiangyong proglacial lake exhibited the highest species diversity and richness, characterized by the overwhelming dominance of Pseudomonadota and the highest number of unique OTUs in water, which together suggest the presence of a more distinct ecological niche (Figure 2 and Figure 3). The water of Gangbugou proglacial lake showed the highest relative abundance of Bacteroidota. In contrast, Actinomycetota was the dominant group in the water of the Qudengnima proglacial lake, which was situated at the highest altitude and has a significantly higher pH than the other two lakes (Figure 1 and Figure 8a). It is likely that stronger ultraviolet radiation and unique environmental conditions exert a selective effect on community assembly in this lake. This interpretation aligns with earlier work in high altitude lakes, where UV radiation has been identified as a dominant selective force that shapes bacterioplankton community composition [42,43]. Regarding habitat differences, as shown in Figure 8a,b, the bacterial community structures in sediments exhibited high similarity across the proglacial lakes, suggesting a relatively stable microenvironment that likely buffered the effects of geographic variation. the bacterial community structures in sediments exhibited higher similarity across the proglacial lakes, suggesting a relatively stable microenvironment that likely buffered the effects of geographic variation. This pattern of sediment communities being more spatially homogeneous than their pelagic counterparts is increasingly recognized in lake ecosystems: Vettorazzo et al. [44] conducted a study on the planktonic and benthic bacterial communities in 19 freshwater bodies in the Alps region. The results showed that water and sediment habitats harbored unique bacterial communities with significant differences in their taxonomic compositions. The benthic bacteria show no significant differences among different types of freshwater. Conversely, planktic communities showed greater heterogeneity, closely mirroring our “convergence in sediments” pattern. Furthermore, Liu et al. [6] demonstrated that there are significant differences between the bacterial communities in the water and sediment of glacial lakes on the Qinghai-Xizang Plateau; the β-diversity of the water bacterial community increased significantly with increasing GI, whereas the β-diversity of the sediment community decreased significantly with increasing GI. The bacterial alpha diversity in lake water increased with increasing glacial index (GI). Contrarily, the alpha diversity of sediment bacterial communities increased with decreasing GI. This implies that sediment bacterial diversity appears less influenced by changes in the external environment [6]. Our results therefore reinforce the emerging consensus that sediment provided a more stable habitat, reducing the geographic imprint on bacterial community structure compared to the dynamic water. In contrast, the water environment of the proglacial lake is more susceptible to disturbances caused by fluctuations in the ambient environment—such as fluctuations in water temperature and thermal structure. Cold glacier meltwater and low nighttime air temperatures caused a distinct diurnal pattern of water temperature in the water column of glacier-influenced lakes. Precipitation onto glacier surfaces apparently leads to rapid cooling of the glacier-fed lakes and disrupts the thermal stratification with several mixing events during the summer [45]. Fluctuations in turbidity and ultraviolet radiation intensity: the glacial meltwater, carrying high concentrations of glacial flour, will make the water of a proglacial lake become turbid. The changes in cloud cover and turbidity cause fluctuations in the intensity of ultraviolet radiation in the water of proglacial lakes [42]. Fluctuations in nutrients and contaminants: the fluctuations in substances such as nitrogen and phosphorus are caused by events like glacial meltwater, atmospheric deposition, and rainfall. Furthermore, in the face of global warming and the consequent accelerated glacier retreat, the inputs of DOM, Hg, and other constituents into proglacial lakes, as well as their potential impacts, are being intensified [46,47]—which likely contributed to the more distinct bacterial community structures observed in the water among the three lakes. The high compositional similarity among sediment communities might also be underpinned by evolutionary mechanisms. The sediment habitat is characterized by prolonged anoxia, low temperature, and recalcitrant carbon inputs. Under such conditions, traits that are deeply conserved in certain microbial lineages become the overriding filters that select for a narrow range of phylogenetic groups. The repeated selection of these phylogenetically cohesive groups across sediment samples could explain the observed low β-diversity. In this view, the higher similarity is not simply a reflection of similar abiotic conditions, but also a signature of evolutionary niche conservatism on the microbial community assembly. Future work incorporating phylogeny-based community assembly analyses (e.g., NRI or βNTI) will allow a direct test of this hypothesis. In summary, the bacterial communities in these three proglacial lakes showed a pattern of convergence in sediments but divergence in water, thereby illustrating the distinct roles that different environmental matrices play in shaping microbial assemblages in proglacial lakes (Figure 8a,b). A similar result was observed in glacial lakes within the forefield catchments of three glaciers in the Eastern Swiss Alps [28]. Despite sediments also receiving glacier snow-derived bacteria in a manner similar to lake water, the average proportion was much lower (2.5% compared to 49.3% in water). This implies that sediment bacterial diversity appears less influenced by inputs of glacial snow-derived species. Studies of glaciers in other regions have also identified Pseudomonadota (Proteobacteria) as a dominant bacterial phylum. Liu et al. [48] found Pseudomonadota to be the predominant phylum in the analysis of bacterial diversity in meltwater from a −183 m deep ice layer of the Dalk Glacier, East Antarctica. Segawa et al. [49] investigated the Yamato and Mizuho glaciers in East Antarctica; their results showed that Gammaproteobacteria (46%) dominated in the Yamato ice core, and that Firmicutes (47%) was the predominant phylum in the Mizuho ice layer. Fan et al. [50] investigated the microbial community structure in soils from Antarctica’s Nelson Glacier, where the predominant bacterial group was identified as Proteobacteria. Similarly, studies of glaciers on the Qinghai-Xizang Plateau have also reported Proteobacteria as a dominant bacterial phylum. Ye et al. [51] investigated the microbial communities in the Azha and Midui glaciers of southeastern Xizang; they found that Proteobacteria (40%), Actinomycetota (23%), and Bacteroidota (14%) were the most abundant phyla. This is consistent with previous reports identifying Proteobacteria, (42.56%), as the dominant bacterial group in Qinghai-Xizang Plateau glaciers. Liu et al. [52] investigated microorganisms in various aquatic systems of the Qinghai-Xizang Plateau; they reported a community primarily composed of Pseudomonadota (31.5%), Bacteroidota (23.3%), Actinomycetota (19.0%), and Verrucomicrobiota (6.2%). These findings are consistent with the results of the present study. A previous study reported nine bacterial phyla with a relative abundance >1% in Qinghai-Xizang Plateau glaciers [52]. This slightly differs from our results. In our study, six phyla in the water and ten phyla in the sediment had relative abundance above 1%. Furthermore, phyla like Cyanobacteriota, Acidobacteriota, and Chloroflexota also showed high relative abundance here (Figure 8a). These differences suggest that microbial communities can vary even within the same regional glacial environment, which may be attributed to local geographic and climatic factors.

Beyond the physicochemical drivers discussed above, several glacier-specific factors collectively shape bacterial communities in these proglacial lakes, thereby offering an integrated perspective on glacier-specific drivers. The distinct bacterial community patterns observed in the three proglacial lakes can be explained by four interconnected glacial environmental factors. First, meltwater hydrology continuously introduces allochthonous bacteria and nutrients from glaciers, with variable input intensity among lakes, contributing to divergent water communities. Second, seasonal turnover (e.g., freeze–thaw cycles) likely affects water more than sediment, as sediments remain thermally buffered; this aligns with our finding that sediment communities converged across lakes, while water communities diverged. Third, sediment particle absorption provides stable attachment surfaces, enhancing diversity and harboring particle-associated taxa such as *Sphingomonas* and Acidobacteriota; the absence of such particles in water explains the lower diversity and higher inter-lake variability in planktonic communities. Fourth, redox gradients differ markedly between water and sediment: water ORP varied significantly among proglacial lakes (highest in Qiangyong), correlating with nitrogen species, whereas sediment redox stratification (coexistence of NH_4_^+^ and NO_3_^−^) supports both nitrifiers and denitrifiers (e.g., Pseudomonadota). Collectively, these four drivers—hydrology, seasonality, particle attachment, and redox heterogeneity—shape the bacterial communities in a habitat-specific manner, accounting for the higher diversity and stability in sediments versus the greater spatial variability in water.

As shown in Figure 3, the alpha diversity indices (species diversity and richness) were higher in sediments than in the water across the study area, this trend is consistent with findings reported from other glacial environments. Tian et al. [53] studied the distribution of culturable bacterial communities on the Musu Island Glacier. They found that species diversity varied greatly among different habitats; the highest species diversity was in forefield soil, while the lowest was in surface meltwater. Ren et al. [54] showed that bacterial communities in glacier forefield soils had higher alpha diversity compared to those in adjacent glacier-fed streams. Similar conclusions have been reported for other aquatic environments. For instance, in sea cucumber aquaculture ponds, Liu et al. [55] found significantly lower alpha diversity in the water than in the sediment. Similarly, Zhang et al. [56] reported that alpha diversity indices in sediments of the Chaobai River were substantially higher than those in water samples. The greater alpha diversity in sediments is likely due to their richer nutrient availability and greater environmental stability compared to the water [57]. Sediments offer a more buffered habitat, with less exposure to light, pollutants, and hydrodynamic disturbances, as well as more stable temperatures, which collectively reduce environmental stress for microorganisms [58]. The alpha diversity results for the three proglacial lakes indicated that Qiangyong exhibited the highest species richness and diversity in both water and sediment. Specifically, the Shannon index in its water was significantly higher than those in Qudengnima and Gangbugou, which is closely associated with its highest total phosphorus content and relatively nutrient-rich conditions (Figure 1 and Figure 3). In contrast, the relatively lower alpha diversity in the water of Qudengnima proglacial lake may be related to the selective pressure exerted by its extreme environment, characterized by high altitude, intense radiation, and high pH (Figure 1 and Figure 3). Beta diversity results (Figure 4) showed that bacterial community composition differed little among sediment samples from the three proglacial lakes, but bacterial species composition differed greatly in the water. Environmental conditions are more stable in sediments, making microbial communities less influenced by geographic location. In contrast, water is more susceptible to external disturbances like temperature, UV radiation, and rainfall. Microbial dispersal is more limited in sediments, whereas high water flow enhances mixing and dispersal. Ren et al. [54] found that bacterial communities in a glacier-fed stream of the Hailuogou Glacier exhibited significantly higher beta diversity than in its forefield soils, indicating greater taxonomic heterogeneity in the stream—a finding consistent with this study. However, inconsistent results also exist. The same study [54] found no significant difference in beta diversity between soils and streams on the Ürümqi Glacier No. 1. Furthermore, Kleinteich et al. [59] observed no significant differences in microbial community composition across three distant glacial catchments. These findings indicate that environmental factors are more important than geographic location in shaping bacterial community composition. In this study, the sediment bacterial communities showed high similarity across the three proglacial lakes, implying a “homogenizing” effect of the sedimentary microenvironment. But the bacterial communities in the water differed significantly, indicating that variations in water chemistry, nutrient levels, and glacial meltwater inputs play a key role in shaping the bacterial assemblages in these proglacial lakes. For example, in the PCoA analysis, the water samples from Qiangyong proglacial lake and Qudengnima proglacial lake were the most distant in community composition (Figure 4a). This pattern corresponded to their significant differences in physicochemical properties such as pH, altitude, and total phosphorus, demonstrating that subtle differences in the aquatic environment are sufficient to drive distinct microbial community structures.

RDA results indicated that pH, total phosphorus, and ammonium nitrogen were the key physicochemical factors influencing the bacterial community structure in water (Figure 6a), while total carbon was the primary driver in sediments (Figure 6b). pH serves as a direct indicator of variations in other environmental factors and can indirectly influence microbial community structure. In high-pH water environments, bacterial community stability tends to decrease. Phosphorus and other nutrients can be utilized as energy sources, and phosphorus availability notably alters microbial acquisition of carbon and nitrogen, thereby affecting microbial composition [60]. Studies of microbial communities in glacial environments have shown that pH, ammonium nitrogen, and total phosphorus are associated with bacterial community composition [6,61], which aligns with the findings of this study. RDA analysis revealed a positive correlation between the bacterial community in the water of Qudengnima proglacial lake and high pH, consistent with the high relative abundance of Actinobacteria, suggesting that alkaline conditions may favor the colonization and growth of this phylum. In contrast, the dominance of Pseudomonadota in the water of Qiangyong proglacial lake was closely linked to significantly elevated total phosphorus levels (Figure 6a), indicating potentially more active phosphorus cycling in this lake. These correlative analyses clarify the key environmental drivers shaping the bacterial communities in each lake. It has also been reported that the composition of bacterial communities in the sediments of alpine lakes is related to parameters such as temperature [59]. Ji et al. [62] stated that temperature played a crucial role in determining the bacterial community structure of mountain stream sediments and had a significant impact on the bacterial metabolic processes. Temperature directly (43.70%) and indirectly (41.10%) affects the bacterial community structure by influencing sediment parameters. However, the impact of temperature on functional composition is not significant. A limitation of this study is the absence of direct in situ sediment temperature measurements, which restricted our ability to fully resolve the effect of temperature on the microbial community. Future work will incorporate sediment temperature as a core environmental variable. A limitation of the current bioinformatics pipeline is the use of QIIME 1.9.1 with 97%–OTU clustering rather than the QIIME 2 framework with ASV-level denoising (DADA2/Deblur), which offers improved error modelling and exact-sequence resolution. While OTU-based approaches remain widely published, future re-analyses will adopt QIIME 2 to enable higher-resolution taxonomic inference and improved low-abundance signal recovery. Additionally, In future research, we will employ metagenomic shotgun sequencing and explore the public health risks issues like human pathogens and animal parasites.

## 5. Conclusions

This study employed 16S *rRNA* gene amplicon sequencing to systematically analyze the diversity and composition of bacterial communities in high-altitude proglacial lakes on the southern Qinghai-Xizang Plateau. The results showed that the abundances of Pseudomonadota, Actinomycetota, and Bacteroidota were particularly prominent. In these three proglacial lakes, the bacterial community structures in the water varied considerably, while those in the sediments were relatively similar. The pH, total phosphorus, and ammonium nitrogen significantly shaped the microbial community structure. These findings provide baseline ecological data for understanding microbial biogeography and community assembly in proglacial lakes on Qinghai-Xizang Plateau. However, this study has some limitations. For example, the use of OTU-based clustering and the QIIME 1.9.1 platform represents a methodological limitation, which may constrain the resolution of taxonomic variants. In the subsequent research, we will adopt ASV-based pipelines, up-to-date bioinformatic tools, and, where feasible, shotgun metagenomic approaches to obtain deeper functional insights. In addition, OTU analysis is suitable for our macro-ecological comparisons but may miss fine-scale genetic variants detectable by ASV methods. Due to computational constraints, re-analysis was not performed. Future work will prioritize ASV-based pipelines.

## Figures and Tables

**Table 1:** Sampling sites information of three proglacial lakes.

Number	Sampling Sites	Altitude/m	Latitude	Longitude	Location
QDNM-1	Site No. 1 of the Qudengnima proglacial lake	5200	28°1′27″ N	88°15′59″ E	Gamba County, Shigatse, Xizang Autonomous Region
QDNM-2	Site No. 2 of the Qudengnima proglacial lake	5200	28°2′39″ N	88°13′05″ E	Gamba County, Shigatse, Xizang Autonomous Region
QDNM-3	Site No. 3 of the Qudengnima proglacial lake	5200	28°0′57″ N	88°15′18″ E	Gamba County, Shigatse, Xizang Autonomous Region
GBG-1	Site No. 1 of the Gangbugou proglacial lake	4900	28°56′07″ N	90°12′07″ E	Nagarzê County, Shannan, Xizang Autonomous Region
GBG-2	Site No. 2 of the Gangbugou proglacial lake	4900	28°57′25″ N	90°11′02″ E	Nagarzê County, Shannan, Xizang Autonomous Region
GBG-3	Site No. 3 of the Gangbugou proglacial lake	4900	28°55′31″ N	90°13′27″ E	Nagarzê County, Shannan, Xizang Autonomous Region
QY-1	Site No. 1 of the Qiangyong proglacial lake	4870	28°52′55″ N	90°14′51″ E	Nagarzê County, Shannan, Xizang Autonomous Region
QY-2	Site No. 2 of the Qiangyong proglacial lake	4870	28°51′28″ N	90°14′10″ E	Nagarzê County, Shannan, Xizang Autonomous Region
QY-3	Site No. 3 of the Qiangyong proglacial lake	4870	28°53′32″ N	90°13′31″ E	Nagarzê County, Shannan, Xizang Autonomous Region

## Data Availability

The original contributions presented in this study are included in the article/Supplementary Materials. Further inquiries can be directed to the corresponding authors.

## Supplementary Materials

The following supporting information can be downloaded at: https://www.mdpi.com/article/10.3390/microorganisms14071398/s1. Figure S1. Physicochemical properties of water in different proglacial lakes. Note: The samples marked with different lowercase letters showed significant differences (*p* < 0.05), and the least significant difference (LSD) test was used. Abbreviations: NO_3_^−^-N, nitrate nitrogen; NO_2_^−^-N, nitrite nitrogen; ORP, oxidation–reduction potential; OC, Organic Carbon; TN, Total Nitrogen; TP, Total Phosphorus; QDNM-W, water samples from Qudengnima proglacial lake; GBG-W, water samples from Gangbugou proglacial lake; QY-W, water samples from Qiangyong proglacial lake. Figure S2. Physicochemical properties of sediment in different proglacial lakes. Note: The samples marked with different lowercase letters showed significant differences (*p* < 0.05), and the least significant difference (LSD) test was used. Abbreviations: NH_4_^+^-N, ammonium nitrogen; EC, Electrical Conductivity; MC, Moisture Content; TP, Total Phosphorus; TS, Total Sulfur; TDS, Total Dissolved Solids; NO_3_^−^-N, nitrate nitrogen; OC, Organic Carbon; TN, Total Nitrogen; TC, Total Carbon; QDNM-S, sediment samples from Qudengnima proglacial lake; GBG-S, sediment samples from Gangbugou proglacial lake; QY-S, sediment samples from Qiangyong proglacial lake. Figure S3. Cladogram generated from the LEfSe analysis. Abbreviations: LEfSe, Linear Discriminant Analysis Effect Size; p, phylum; c, class; o, order; f, family; g, genus; QDNM-W, water samples from Qudengnima proglacial lake; GBG-W, water samples from Gangbugou proglacial lake; QY-W, water samples from Qiangyong proglacial lake; QDNM-S, sediment samples from Qudengnima proglacial lake; GBG-S, sediment samples from Gangbugou proglacial lake; QY-S, sediment samples from Qiangyong proglacial lake.
