# Supplementary material for: Diversity and Community Structure of Bacteria in High-Altitude Proglacial Lakes in Southern Qinghai-Xizang Plateau
Source: Microorganisms. 2026 Jun 24;14(7):1398. doi: 10.3390/microorganisms14071398 (PMC13413640; doi:10.3390/microorganisms14071398)
Supplement: Supplementary file 1 [file microorganisms-14-01398-s001.zip › microorganisms-4319365-supplementary.pdf]

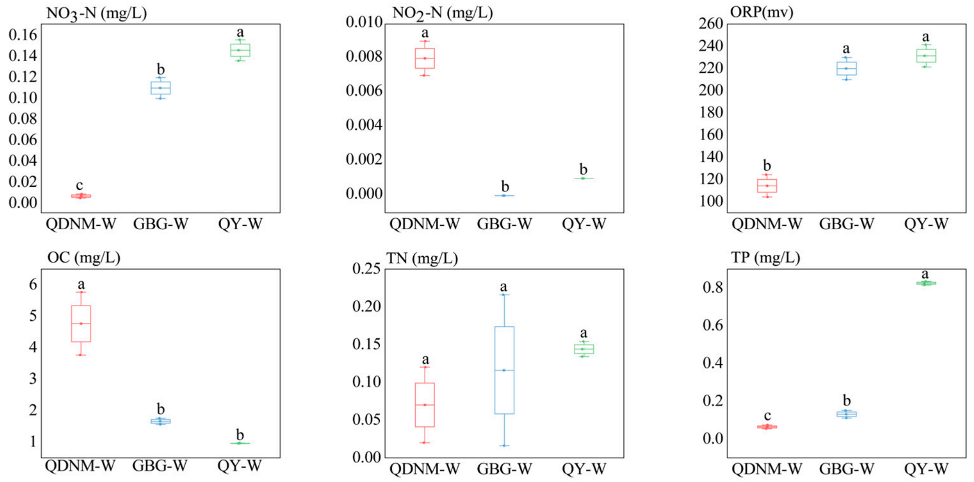

Figure S1. Physicochemical properties of water in different proglacial lakes. Note: The samples marked with different lowercase letters showed significant differences (p < 0.05), and the least significant difference (LSD) test was used. Abbreviations: NO_3^--N, nitrate nitrogen; NO_2^--N, nitrite nitrogen; ORP, oxidation–reduction potential; OC, Organic Carbon; TN, Total Nitrogen; TP, Total Phosphorus; QDNM-W, water samples from Qudengnima proglacial lake; GBG-W, water samples from Gangbugou proglacial lake; QY-W, water samples from Qiangyong proglacial lake.

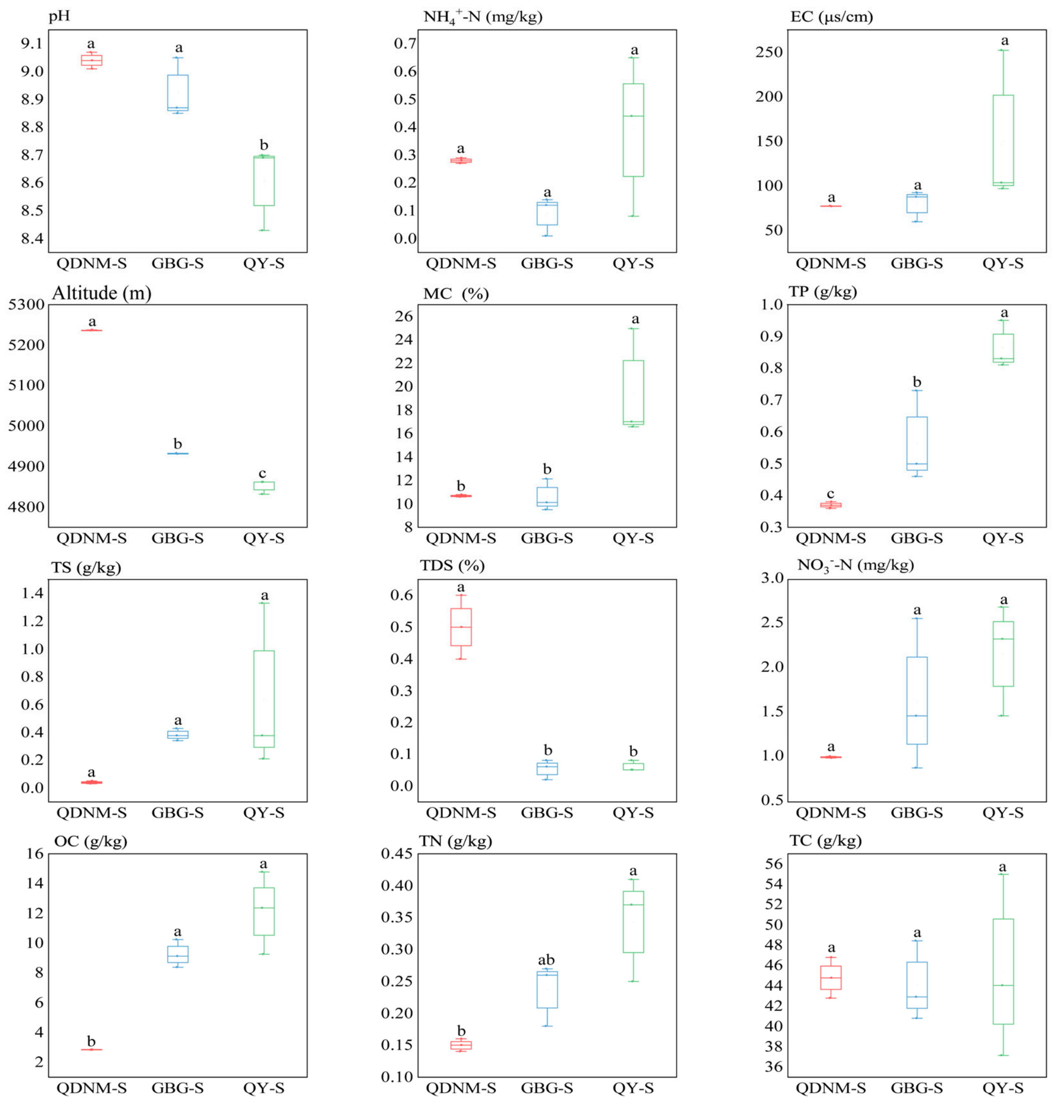

Figure S2. Physicochemical properties of sediment in different proglacial lakes. Note: The samples marked with different lowercase letters showed significant differences ($p < 0.05$), and the least significant difference (LSD) test was used. Abbreviations: NH_4^+-N, ammonium nitrogen; EC, Electrical Conductivity; MC, Moisture Content; TP, Total Phosphorus; TS, Total Sulfur; TDS, Total Dissolved Solids; NO_3^--N, nitrate nitrogen; OC, Organic Carbon; TN, Total Nitrogen; TC, Total Carbon; QDNM-S, sediment samples from Qudengnima proglacial lake; GBG-S, sediment samples from Gangbugou proglacial lake; QY-S, sediment samples from Qiangyong proglacial lake.

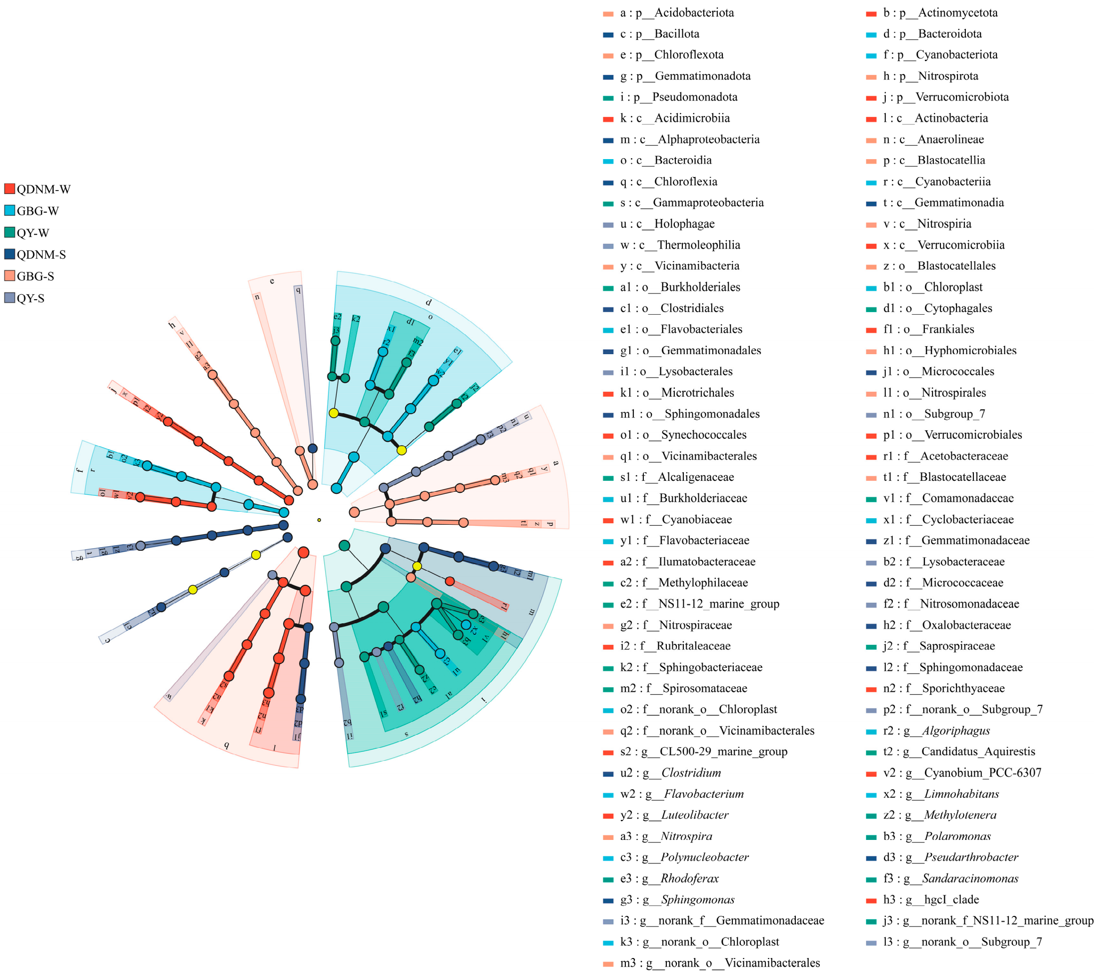

Figure S3. Cladogram generated from the LEfSe analysis. Abbreviations: LEfSe, Linear Discriminant Analysis Effect Size; p, phylum; c, class; o, order; f, family; g, genus; QDNM-W, water samples from Qudengnima proglacial lake; GBG-W, water samples from Gangbugou proglacial lake; QY-W, water samples from Qiangyong proglacial lake; QDNM-S, sediment samples from Qudengnima proglacial lake; GBG-S, sediment samples from Gangbugou proglacial lake; QY-S, sediment samples from Qiangyong proglacial lake.
